# Prenatal Diagnosis of Fryns Syndrome through Identification of Two Novel Splice Variants in the *PIGN* Gene—A Case Series

**DOI:** 10.3390/life14050628

**Published:** 2024-05-14

**Authors:** Aruna Marchetto, Susanne Leidescher, Theresia van Hoi, Niklas Hirschberger, Florian Vogel, Siegmund Köhler, Ivonne Alexandra Bedei, Roland Axt-Fliedner, Moneef Shoukier, Corinna Keil

**Affiliations:** 1 Eurofins Humangenetik und Pränatal-Medizin MVZ GmbH, 80639 Munich, Germanyshoukier@praenatal-medizin.de (M.S.); 2 Department of Prenatal Medicine and Fetal Therapy, Philipps University, 35043 Marburg, Germany; siegmund.koehler@med.uni-marburg.de; 3 Department of Prenatal Medicine and Fetal Therapy, Justus-Liebig University Giessen, 35392 Giessen, Germany

**Keywords:** *PIGN*, Fryns syndrome, prenatal diagnosis, RNA sequencing

## Abstract

Fryns syndrome (FS) is a multiple congenital anomaly syndrome with different multisystemic malformations. These include congenital diaphragmatic hernia, pulmonary hypoplasia, and craniofacial dysmorphic features in combination with malformations of the central nervous system such as agenesis of the corpus callosum, cerebellar hypoplasia, and enlarged ventricles. We present a non-consanguineous northern European family with two recurrent cases of FS: a boy with multiple congenital malformations who died at the age of 2.5 months and a female fetus with a complex developmental disorder with similar features in a following pregnancy. Quad whole exome analysis revealed two likely splicing-affecting disease-causing mutations in the *PIGN* gene: a synonymous mutation c.2619G>A, p.(Leu873=) in the last nucleotide of exon 29 and a 30 bp-deletion c.996_1023+2del (NM_176787.5) protruding into intron 12, with both mutations in *trans* configuration in the affected patients. Exon skipping resulting from these two variants was confirmed via RNA sequencing. Our molecular and clinical findings identified compound heterozygosity for two novel splice-affecting variants as the underlying pathomechanism for the development of FS in two patients.

## 1. Introduction

The prenatal diagnosis of congenital anomaly syndromes is a major challenge both for the professional team and the parents of the affected child. Genetic testing is crucial in identifying the causes of the current clinical picture in these cases; providing a prognosis for the child is essential when counselling parents. However, the evaluation of extremely rare phenotypes by comprehensive sequencing methods has proven difficult in terms of interpreting the clinical significance of variants and proving their causality. Furthermore, molecular genetic studies of complex diseases are complicated by the heterogeneity of the diseases and the different underlying pathomechanisms. Among these, splicing anomalies pose a particular challenge, as changes in splicing-relevant domains are often difficult to predict and evaluate.

Splicing relies on consensus sequences, so called “splice sites“, located at both ends of an intron as well as “branch points“ located upstream of the 3‘ end of an intron that serve as anchors and processing signals for the splicing machinery (“spliceosome“) [1].

While in some cases alternative splicing is needed to produce different isoforms of a protein (e.g., titin isoforms in skeletal muscle and heart muscle), aberrant splicing can severely affect protein function [2,3]. In particular, if a conserved base or sequence involved in the orchestration of the splicing reaction is altered, this may have far-reaching consequences leading to altered non-intended transcripts, and consequently, altered or truncated proteins not able to properly execute their intended function [4].

In this article, we describe two cases of Fryns syndrome with lethal course within one family. In the process of molecular diagnostic testing, two novel genetic variants affecting splicing in the *PIGN* gene were detected in both affected individuals. Our findings not only describe two previously unknown splice affecting variants in the *PIGN* gene in the context of FS, but also emphasize the advantages of genetic analyses at the RNA level and illustrate the importance of interdisciplinary approaches in linking clinical phenotypes to potential underlying genetic variants. We anticipate that the presented work will be of great benefit for the interpretation of splicing-relevant *PIGN* variants in the context of FS.

### 1.1. Fryns Syndrome (FS)

FS is one of the most common syndromes associated with congenital diaphragmatic hernia (1.3–10%) and has an incidence of 1/14,000 births [5,6,7]. A total of 76–89% of all FS cases are associated with congenital diaphragmatic hernia (CDH), and autosomal recessive inheritance has long been assumed due to the occurrence of several cases in one family [8,9]. Since the majority of affected children do not survive the neonatal period, the course was long considered lethal [10,11]. However, 14% of the reported cases survived the neonatal period, particularly in the absence of CDH and pulmonary hypoplasia, fewer complex cardiac malformations, and less neurological deficits [9]. 

In 2004, Slavotinek described FS with the following characteristics, based on 52 reported cases: (1) hydramnios, (2) a characteristic facial appearance (coarse face, hypertelorism, a broad and flat nasal bridge with thick tip, long philtrum, low-set and poorly formed ears, a tented upper lip with macrostomia and micrognathia, (3) pulmonary hypoplasia, (4) distal digital hypoplasia, and (5) internal malformations including the characteristic diaphragmatic hernia as well as gastrointestinal, genitourinary, and cardiovascular malformations [12]. Overall, the diagnosis of FS is difficult due to overlap with a variety of syndromes with variable clinical presentation including Trisomy 22 and Pallister–Killian syndrome [13,14]. 

In 2005, Lin et al. published a scheme that defined FS and is intended to facilitate its differentiation from similar phenotypes. They proposed a classification in which four of the six major features are required to define FS (Table 1); if three features are present, a “Fryns-like phenotype” is assumed [15]. 

Kosinski et al. proposed a distinction between FS, which follows autosomal recessive inheritance and an Fryns-like phenotype, which has the clinical features of FS but may have different molecular genetic causes, supporting the classification published by Lin et al. [15,16].

As FS is a complex and heterogeneous malformation syndrome with high morbidity and mortality, this highlights the necessity for a thorough prenatal diagnosis. 

### 1.2. Genetic Findings in the Context of FS

An autosomal recessive pattern of inheritance for FS was suggested early on due to the occurrence in families with consanguineous parents and affected siblings [17,18,19]. Further case reports confirmed the autosomal recessive character without consanguineous parents [20].

Several case studies have described biallelic (for example in the *PlGN*, *PIGW*, *PIGV*, *PIGS* genes) or hemizygous (*PlGA* gene) variations in genes involved in the glycosylphosphatidylinositol (GPI)-anchor biosynthesis pathway in combination with the FS or Fryns-like phenotype [21,22,23,24,25]. In general, biallelic (or hemizygous) pathogenic variants in genes coding for proteins involved in the pathway of building GPI-anchored proteins (GPI-APs) result in defects summarized as GPI biosynthesis defects (GPIBDs). Defective GPI-APs primarily impair signal transduction and cell adhesion and GPIBDs show different partially overlapping symptoms often including seizures, congenital (dysmorphic) anomalies, and intellectual disability [26]. Mutations that disrupt the formation of GPI-AP such as *PIGN* mutations result in an overall reduction in functional GPI-anchored proteins including, for example, cluster of differentiation (CD) molecules. Biallelic variants in the *PIGN* gene cause both FS and multiple congenital anomalies, hypotonia, and seizures syndrome 1 (MCAHS1) [27]. However, FS tends to present with more severe symptoms than MCAHS and is typically associated with the presence of a congenital diaphragmatic hernia. 

### 1.3. Case Presentation

#### 1.3.1. Case 1

A non-consanguineous northern European couple, a 27-year-old female (BMI 20) and a 25-year-old male, presented at our tertiary center at 34.4 weeks gestational age (GA) with hydramnios of unknown origin. There was a history of four early miscarriages and one unremarkable pregnancy with a healthy child. No other relevant family history was reported.

First trimester screening described nuchal translucency > 95th percentile and led to amniocentesis, which could not be evaluated due to a lack of cell growth. No further diagnostics were performed, and pregnancy was continued. Further scans did not identify any pathological findings. A symptomatic hydramnion quickly developed from the 30-week GA on, leading to a presentation at our center.

The initial examination revealed hydramnios (single deepest pocket 13 cm), a large for gestational age (LGA) fetus (>95th percentile) with unilateral pelvic kidney, hydronephrosis °3, megaureter, and renal agenesis of the opposite side. In addition, a diagnosis of aortic valve stenosis with myocardial hypoplasia and subaortic ventricular septal defect (VSD) was suspected. The fetus presented a generalized skin edema, cranio-fascial malformation, and a singular umbilical artery; fetal–maternal Doppler was unremarkable. The parents declined another amniocentesis. Following multidisciplinary counselling, the parents decided to continue the pregnancy. At 39.0 weeks GA, a boy was born via caesarean section due to obstetric reasons. Postnatal examination confirmed renal and cranio-facial malformation (coarse face, cleft of lip and palate), and further malformations were diagnosed: corpus callosum agenesis, narrow thorax, widely spaced nipples, duodenal atresia, hepatomegaly. No cardiovascular malformation and no CDH was found (Table 1).

An exploratory laparotomy was performed due to suspected duodenal stenosis and revealed duodenal non-rotation. At discharge, the boy had intermittent seizures, severe muscular hypotonia with opisthotonos, and marked weakness in drinking and swallowing in association with an underlying syndromic disease. At the age of 2.5 months, the boy was readmitted to hospital due to severe pneumonia. He quickly developed respiratory failure and died in status epilepticus within a few days after admission. Autopsy was offered due to the multiple malformations, which appeared to be caused by a congenital syndromic disease, but was refused. 

Due to the history of four early miscarriages and the lethal course of the pregnancy, the parents were informed about the possibility of human genetic counselling.

#### 1.3.2. Case 2

In the following pregnancy (1.5 years later), the couple presented at 14.4 weeks GA with generalized cutaneous edema, cystic nuchal hygroma, and left-sided CDH. No additional genetic testing had been performed in between the pregnancies. The patient underwent amniocentesis at 16.6 weeks GA, which revealed a normal female karyotype and array-CGH.

Further sonographic follow-up revealed normal fetal growth (50th percentile according to Hadlock) but multiple anomalies: cranio-facial malformation, a cleft palate, truncus arteriosus communis with accompanying VSD, symmetrical shortening of all long bones, generalized cutaneous edema (without hydrops fetalis), and a high-grade suspicion of a gyration disorder combined with corpus callosum agenesis. Parents were counseled by a multidisciplinary team (pediatric, pediatric-surgery, human genetics, obstetric) and decided to continue with the pregnancy. Due to symptomatic hydramnios (abdominal pressure and tension, premature contraction), uncomplicated amnion reduction (1000 mL) was performed twice during further pregnancy course (30th and 35th weeks GA) and led to an improvement in maternal symptoms.

Caesarean section was performed for maternal indication at 37.3 weeks. A total of 8 L of amniotic fluid was drained; no severe uterine bleeding occurred during surgery and perioperative blood loss was approximately 400 mL. There were no further maternal complications (postpartum hemorrhage) during the admission.

The female newborn showed facial malformation (coarse face) including cleft of lip and palate, narrow thorax, widely spaced nipples, and the left CDH. Cardiac examination confirmed truncus arteriosus communis °3 and VSD; the abdominal scan showed hepatomegaly. Hypoplastic nails were found on the fingers, while feet showed absent nails. The infant presented muscular hypotonia and multiple contractions in the upper and lower limbs. Sonographic examination of the brain demonstrated abnormal development of the sulci and gyri. The girl died within the first day of life after the exhaustion of intensive medical measures. Post-mortem examination was declined by the parents. 

After experiencing the loss of two children and multiple early miscarriages, the parents consented to further genetic testing after thorough multidisciplinary counselling. The analysis included the mother’s and father’s blood as well as material from the amniocentesis performed on each child.

## 2. Materials and Methods

### 2.1. Whole Exome Sequencing (WES) 

Genomic DNA extraction from EDTA blood and amniocytes was carried out by using the QIAamp DNA Blood Mini Kit (Qiagen, Hilden, Germany) according to the manufacturer’s protocol. The concentration of DNA was determined by using the Qubit system (ds DNA Assay Kit; Thermo Fisher Scientific, Waltham, MA, USA). Whole exome sequencing was performed by using an Illumina Twist Exome 2.0 Plus (Illumina, San Diego, CA, USA) according to the manufacturer’s protocol. By using the hybrid capture method, genomic DNA was processed, with relevant regions enriched and amplified by PCR (Illumina^®^ DNA Prep with Exome 2.0 Plus Enrichment, San Diego, CA, United States). Massively parallel sequencing was performed using a NextSeq2000 Sequencing System (Illumina, San Diego, CA, USA). WES quality criteria required a minimal coverage of 30-fold for 100% of the investigated target regions. More detailed information on the protocols used is available upon request.

### 2.2. RNA Sequencing

Blood was collected in PAXgene blood RNA tubes (BD Biosciences, Franklin Lakes, NJ, USA). RNA was extracted from 2.5 mL whole blood using the NucleoSpin RNA Blood Kit according to the manufacturer’s instructions (support protocol for RNA isolation from whole blood; Macherey-Nagel, Düren, Germany). The concentration of RNA was determined by using the Qubit system (RNA High Sensitivity Assay Kit; Thermo Fisher Scientific, Waltham, MA, USA). RNA sequencing was performed using an Illumina Twist Exome 2.0 Plus on a NextSeq2000 Sequencing System (Illumina, San Diego, CA, USA) according to the manufacturer’s protocols. More detailed information on the protocols used is available upon request.

### 2.3. Bioinformatic Analysis

Fastq generation was executed with the Local Run Manager Generate FASTQ analysis module v2.0. (Illumina, San Diego, CA, USA). For visualization of the identified SNVs and splicing alterations, FASTQ files of the patients’ samples were loaded into the SeqPilot SeqNext module (v5.0, JSI medical systems, Kippenheim, Germany). Exon skipping was detected and visualized with the Fusion Gene Analysis Tool of the SeqPilot SeqNext module (v5.0, JSI medical systems, Kippenheim, Germany). 

Variant classification was carried out according to the ACMG guidelines [28].

## 3. Results

### 3.1. Molecular Analysis

#### 3.1.1. Whole Exome Sequencing (WES)

**Figure 1:**
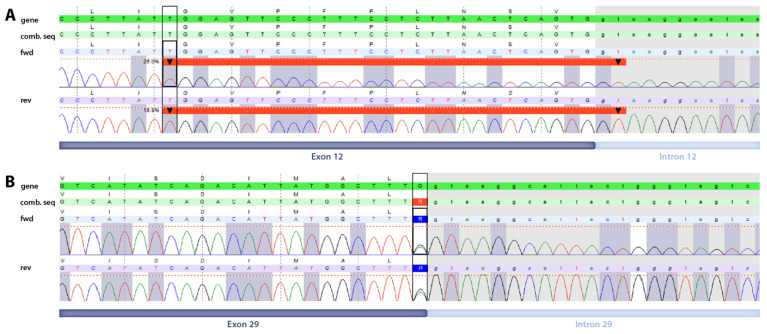
Visualization of the genetic variants detected by WES. (**A**) The position of the 30 bp-deletion *PIGN*:c.996_1023+2del protruding into intron 12 is depicted by a thick red line (start and end positions of the deleted region are indicated by the black downward arrowheads). (**B**) The position of the synonymous variant *PIGN*:c.2619G>A, p.(Leu873=) affecting the last nucleotide of exon 29 is indicated by a black box. Corresponding exon/intron positions are depicted below the images. fwd = forward sequence, rev = reverse sequence, comb. seq = combined sequence (fwd + rev).

The quad whole exome analysis, comprising case 1 and case 2 as well as both parents, revealed two novel compound-heterozygous variants in the *PIGN* gene of the affected probands: the synonymous variant c.2619G>A, p.(Leu873=) affecting the last nucleotide of exon 29 (maternally inherited), and the 30 bp-deletion c.996_1023+2del encompassing a part of exon 12 and two bases of the flanking intron 12 sequence (paternally inherited) (Figure 1). 

#### 3.1.2. Confirmation of Exon Skipping

**Figure 2:**
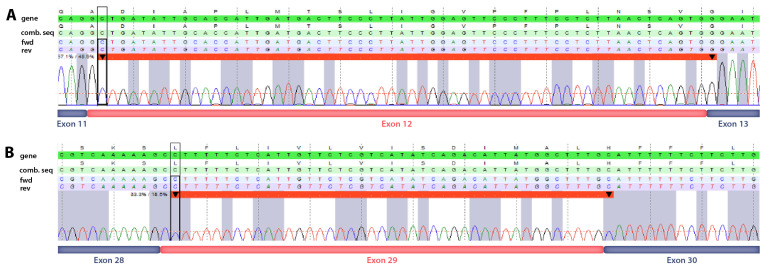
Visualization of exon skipping detected by RNA sequencing in the parents of the affected individuals. (**A**) Skipping of exon 12 (paternal). (**B**) Skipping of exon 29 (maternal). In both cases, the deleted sequence is indicated by a thick red line with start and end positions marked with the black downward arrowheads. Corresponding exon positions are depicted below the images. Note that there was a shift by one base at the position of the exon boundaries in the sequence annotation. This is an alignment artefact, as the corresponding sequences were aligned from the 5’ end. fwd = forward sequence, rev = reverse sequence, comb. seq = combined sequence (fwd + rev).

Gel electrophoresis on cDNA indicated the skipping of exon 12 in the paternal and skipping of exon 29 in the maternal sample [29]. Subsequently, we performed RNA sequencing, which has been proven to be highly effective in detecting aberrant splicing [30], in both parents. RNA sequencing analysis confirmed aberrant transcription of the *PIGN* gene: the paternal sample, harboring the 30 bp-deletion splicing variant c.996_1023+2del, showed the skipping of exon 12 (r.1381_1440del), while the maternal sample, harboring the synonymous variant c.2619G>A, p.(Leu873=), showed the skipping of exon 29 (r.2994_3036del) (Figure 2). 

**Figure 3:**
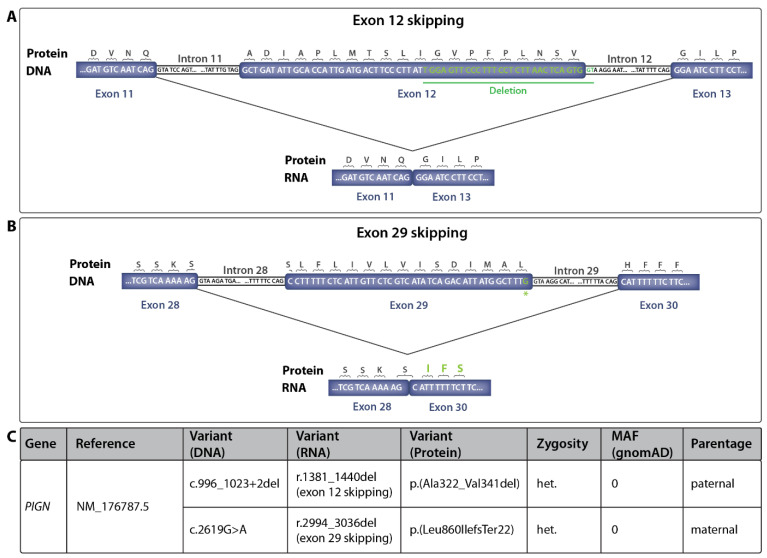
Predicted model for the skipping of exon 12 (**A**) and exon 29 (**B**) based on WES and the RNA sequencing results and protein sequence prediction as well as the proposed resulting protein sequences due to mutations c.996_1023+2del and c.2619G>A, p.(Leu873=), respectively. Sequence alterations detected by WES and the predicted resulting alterations in protein sequence are highlighted in green. (**C**) Table of detected heterozygous splice-affecting alterations in the *PIGN* gene, resulting RNA, and predicted protein alterations. Alterations are described according to human genome variation society (HGVS) regulations [32]. MAF = minor allele frequency, het. = heterozygous.

According to the protein sequence prediction tool ExPASy, the skipping of exon 12 results in the loss of the 20 amino acids encoded by exon 12, but does not alter the reading frame [31]. In contrast, the skipping of exon 29 results in a frameshift mutation, which, according to the prediction tool, leads to alteration of the downstream amino acid sequence and the emergence of a premature stop codon (Figure 3). WES in combination with analysis on the RNA level identified two novel splice affecting variants in the *PIGN* gene in *trans* position in the two siblings affected by FS.

## 4. Discussion

FS is a multiple congenital anomaly syndrome that was first described in 1979 [10]. Characteristically, it is associated with different malformations in combination with neurodevelopmental delay. Pathogenetic findings include hydramnios, CDH with pulmonary hypoplasia, craniofacial dysmorphic features, cleft lip/palate, brachytelephalangy with nail hypoplasia, and various possible other malformations [33]. Even though a definitive genotype–phenotype correlation has not been observed yet, biallelic-truncating variants in the *PIGN* gene usually result in a more severe FS phenotype [27]. In both patients described here, the clinical diagnosis of FS was established according to Lin’s classification, where each case featured five or six of the major characteristics of FS (Table 1) [15].

Although FS is associated with CDH in the majority of cases, there are cases described in the literature without CDH. It has been postulated that the absence of CDH and the related reduced rate of pulmonary hypoplasia is associated with longer survival [34,35]. In line with this, the son who was not affected by CDH (case 1) survived longer than the daughter affected by CDH (case 2).

Due to the severity of the phenotype in both patients, biallelic truncating variants in the *PIGN* gene were assumed. However, a combined effort using Quad-WES and RNA sequencing showed that the genetic alterations in the presented cases of FS were not two but only one truncating variant. On the maternal allele, the skipping of exon 29 presumably led to the emergence of a premature stop codon and therefore to a truncated protein, according to a protein sequence prediction tool. Skipping of exon 12 on the paternal allele, in contrast, led to an in-frame deletion without premature termination of protein biosynthesis. Alessandri et al. reported a deletion in the *PIGN* gene as a founder variant in the La Réunion Island population in patients with FS (c.329_549+1907del5064) [36]. Nevertheless, in this case, a larger deletion spanning from within exon 5 to within exon 7 was detected in a homozygous state in the affected individuals. Furthermore, Brady et al. reported a homozygous donor splice site variant resulting in the skipping of exon 17 of the *PIGN* gene, leading to a premature stop codon in a fetus with bilateral diaphragmatic hernia and multiple congenital anomalies [23]. The synonymous variant c.2619G>A, p.(Leu873=), in contrast, affected one base upstream of the very highly conserved donor splice site sequence “GT”. However, this base was also rather conserved, and thus a noticeable impact, as shown in the present case, can be expected [37]. Similarly, Ohba et al. reported the synonymous alteration c.963G>A, p.(Gln321=) in the *PIGN* gene, resulting in aberrant splicing and the emergence of two transcripts with premature termination codons in the context of congenital anomalies, developmental delay, hypotonia, and epilepsy [38].

In accordance with the literature describing cases of FS with high inter- and even intrafamilial variability, the siblings in the present report also showed partly different symptoms [20,36]. The cases differed in terms of the presence of CDH and a cardiac malformation, resulting in a more rapid lethal course in case 2 (Table 1). 

In addition to the described genetic changes in the *PIGN* gene, other factors that influence the expressivity of the individual phenotypic traits such as other (yet unknown) genetic, epigenetic, or non-genetic factors also potentially influence the phenotypic variability. Although the effects of the genetic changes detected in the present cases could be described by detecting the skipping of exon 12 and exon 29 at the RNA level, it remains generally difficult to predict the effects of splice site affecting genetic alterations. The main reason for this is that these changes can have complex consequences such as the activation of cryptic splice sites leading to (partial) exon skipping or (partial) intron retention. Bioinformatic tools for the assessment of possible splice site alterations can provide a useful resource for assessment. Since the parents did not agree to a post-mortem examination of the girl (case 2), it is also possible that some phenotypic characteristics of the child could not be diagnosed. 

It is important to emphasize that in the present report, RNA analysis helped to understand the molecular genetic changes underlying these severe cases of FS. In the future, it will be essential to incorporate RNA sequencing into molecular genetic workflows to validate potential splice-affecting mutations to assess and contextualize the impact of the mutations on the resulting transcripts. Multi-omics approaches, which in addition to a combination of exome/genome and transcriptome analysis also include the analysis of the proteome and/or epigenome, will continue to significantly improve the diagnostic yield in the future, presumably not least through a better understanding and classification of the splicing alterations.

Our case series shows that not only biallelic truncating variants, but also exon skipping variants, of which in the presented cases one did not lead to premature translation termination, may be relevant in the molecular diagnosis of FS. Furthermore, the present report emphasizes the necessity of interdisciplinary approaches to link clinical phenotypes to possible underlying genetic variants and identified two novel splice affecting mutations in the *PIGN* gene in the context of FS. 

We presented a small case series to provide a significant contribution to our understanding of FS. However, case series are limited through potential bias caused by incomplete data, a lack of comparison groups, and personal opinion. However, they are of crucial importance for understanding rare conditions and their progression; particularly in the case of rare diseases with heterogeneous manifestations, case series/case reports allow for the depiction of individual courses that cannot be represented in larger groups.

The presented case series illustrates the difficulties that exist between prenatal findings and the final diagnosis. Both cases exhibited identical features, but displayed disparate manifestations, complicating the process of reaching a final diagnosis. Despite the close interdisciplinary cooperation, the diagnosis of FS could only be made after the death of both children. However, the late diagnosis provided the parents with a better understanding of their children’s disease. In particular, in patients with syndromic CDH, FS, or FS-like syndrome, a systematic evaluation for variants in the PIGN gene should be considered, as proposed by other groups [34,36]. This approach should be integrated into prenatal and neonatal counseling, particularly in the context of syndromic diseases and/or a suspect family history or consanguineous couples. It is recommended that the interdisciplinary discussion and genetic diagnosis, which goes beyond the karyotype, be established as a fixed component of the prenatal and neonatal counselling process. The high mortality rate resulting from FS, the four early miscarriages in the reported family, and the resulting physical impairment for the mother underscore the need for early genetic diagnosis in further pregnancies. Although genetic counseling only allows for the recurrence risk to be determined, this might be an important aspect for the parents regarding another pregnancy. 

## Figures and Tables

**Table 1:** Comparison of pre- and postnatal characteristics of the siblings (**left**). According to the classification described by Lin et al. (**right**) ≥ 4 out of 6 features must be present to define FS in the clinical context [15]. Based on this classification, the colored columns show the respective characteristics of the two cases. P = percentile, CDH = congenital diaphragmatic hernia, VSD = ventricular septal defect.

	Case 1	Case 2	Lin et al. 2005: FS Defined at Least ≥ 4/6 Features in One Patient
**prenatal**	hydramnios	hydramnios	**(1) Diaphragm defect**
	cutan edema (non hydropic) large for gestational age (> 95. P) nuchal tranlucency > 95. P	cutan edema (non hydropic) 50. P nuchal tranlucency > 95. P	hernia, any location (congenital diaphragmatic hernia, CDH) eventration significant hypoplasia/agenesis
		cleft lip+palate right+left	**(2) Characteristic facial appearance**
	single umbilical artery aortic stenosis ventricular septal defect (VSD)	single umbilical artery Truncus art. com °III ventricular septal defect (VSD)	coarse face, hypertelorism a broad and flat nasal bridge with thick tip, long philtrum low-set and poorly formed ears, a tented upper lip with macrostomia, micrognathia
	no congenital diaphragmatic hernia	left congenital diaphragmatic hernia	
	renal agenesis left hydronephrosis + pelvic kidney right	no renal malformations	**(3) Distal digital hypoplasia**
			nails and/or phalanges
**postnatal**	Corpus callosum agenesis	abnormal development of sulci and gyri	**(4) Significant pulmonary hypoplasia**
	coarse face broard flat nasal bridge cleft lip+palate right + left	coarse face broard flat nasal bridge cleft lip+palate right + left	**(5) Characteristic associated anomalies: At least one**
			Polyhydramnios cloudy cornea and/or microphthalmia orofacial cleft brain malformation cardiovascular malformation renal dysplasia, cortical cysts gastrointestinal malformation genital malformation
	narrow thorax, widely spaced nipples	narrow thorax, widely spaced nipples	
	no cardio-vascular-malformation	Truncus art. communis °3 + VSD	
	no left congenital diaphragmatic hernia	left congenital diaphragmatic hernia	
	no pulmonray hypoplasia	significant pulmonary hypoplasia	
	duodenal atresia		
	hepatomegaly	hepatomegaly	
	renal agenesis left hydronephrosis + pelvic kidney right		**(6) Sibling affected with Fryns syndrome (ideally, diagnosed independently)**
	microgenital		
	n.a.	hypoplastic nails finger absent nails toes	
	muscular hypotonia opisthotonos, seizures	muscular hypotonia multiple contractions upper/lower limbs	

## Data Availability

The data used to support the findings in this study are available in anonymized form from the corresponding author upon reasonable request.
